# Associations between Vitamin D Status, Supplementation, Outdoor Work and Risk of Parkinson’s Disease: A Meta-Analysis Assessment

**DOI:** 10.3390/nu7064817

**Published:** 2015-06-15

**Authors:** Liang Shen, Hong-Fang Ji

**Affiliations:** Shandong Provincial Research Center for Bioinformatic Engineering and Technique, School of Life Sciences, Shandong University of Technology, Zibo 255049, China; E-Mail: shen@sdut.edu.cn

**Keywords:** Parkinson’s disease, vitamin D, outdoor work, meta-analysis

## Abstract

The present study aimed to quantitatively assess the associations between vitamin D and Parkinson’s Disease (PD) risks, which include: (i) risk of PD in subjects with deficient and insufficient vitamin D levels; (ii) association between vitamin D supplementation and risk of PD; and (iii) association between outdoor work and PD risk, through meta-analyzing available data. An electronic literature search supplemented by hand searching up to March 2015 identified seven eligible studies comprising 5690 PD patients and 21251 matched controls. Odds ratio (OR) and 95% confidence interval (CI) of PD risk were assessed through pooling the collected data from eligible studies using Stata software. Pooled data showed that subjects with deficient and insufficient vitamin D levels had increased PD risks compared with matched-controls according to the corresponding OR: 2.08, 95% CI: 1.63 to 2.65, and 1.29, 95% CI: 1.10 to 1.51. Vitamin D supplementation was associated with significantly reduced risk of PD (OR: 0.62, 95% CI: 0.35 to 0.90). Outdoor work was also related to reduced risk of PD (OR: 0.72, 95% CI: 0.63 to 0.81). The findings may stimulate larger, well-designed studies to further verify the associations between vitamin D and PD risk.

## 1. Introduction

Vitamin D is a fat-soluble steroid hormone, which is essential to maintain human health. In recent years, vitamin D has gained tremendous amount of attention owing to the reported associations between low vitamin D level and a wide range of chronic diseases, as well as the therapeutic potential of vitamin D supplementation for these diseases [1,2,3,4,5].

Parkinson’s disease (PD) is the second most common neurodegenerative disorder in elders characterized by dysfunction and degeneration of dopaminergic neurons and the development of intraneuronal inclusions known as Lewy bodies [6,7]. The pathogenetic mechanism underlying PD is complex and remains far to be elucidated, while it is widely accepted that multiple genetic and environmental factors contribute to the development of PD [8,9]. Low vitamin D level has been found in patients with PD in recent years. Since the first study by Sato *et al.* in 1997 [10], many studies found that PD patients had lower vitamin D level compared with controls, and the lower level of vitamin D than controls has been further comprehensively evaluated by meta-analyses [11,12,13]. In the present study, we attempted to quantitatively assess whether low vitamin D level predicts increased PD risk and the association of vitamin D supplementation and PD risk through meta-analyzing available data published so far. As outdoor occupation can optimize vitamin D level due to exposure to solar ultraviolet B radiation, the association between outdoor work and PD risk was also investigated. The findings will provide important clues to the prevention of PD.

## 2. Methods

### 2.1. Literature Search and Inclusion Criteria

We performed a literature search in the MEDLINE database from inception until March 2015 by using the following search terms: “Parkinson’s disease” and “vitamin D” or “25(OH)D”. To identify further potentially relevant studies missed by the search strategy, the reference lists of retrieved articles were also manually screened. A restriction to reference on human studies and those published in English is imposed during reference selection. The potentially eligible references were identified by reviewing titles and/or abstracts, and/or full text of all references obtained with literature search. The inclusion criteria were as follows: (i) original studies investigated the associations of vitamin D level or vitamin D supplementation or outdoor work with PD risk; (ii) the odds ratios (ORs) or relative risks (RRs) with 95% confidence intervals (CI) of PD risk were provided or could be calculated.

### 2.2. Data Extraction and Statistical Analysis

Using prespecified data extraction tables, we extracted information from each eligible article about the study design, main characteristics of the study population, OR or RR and 95% CI of PD risk, and confounding factors that were adjusted during the analysis. If a study provided unadjusted and adjusted OR or RR, the most completely adjusted one was employed. Vitamin D deficiency and insufficiency were defined as serum 25-hydroxyvitamin D [25(OH)D], a stable marker of vitamin D status, concentrations of <50 nmol/L and <75 nmol/L, respectively [14]. Serum concentrations of 25(OH)D reported in ng/mL were converted to values in nmol/L by the conversion factor (1 ng/mL = 2.5 nmol/L). Two reviewers extracted the data from each study independently and consensus was obtained for the extracted data. Based on the extracted data, the OR and 95% CI of PD risk were calculated using the statistical software Stata version 12.0 for windows (StataCorp, College Station, TX, USA). All statistical analyses were performed using the random effect model and the statistical heterogeneity was evaluated through the *I*² statistic.

## 3. Results

### 3.1. Results of the Literature Search

**Figure 1:**
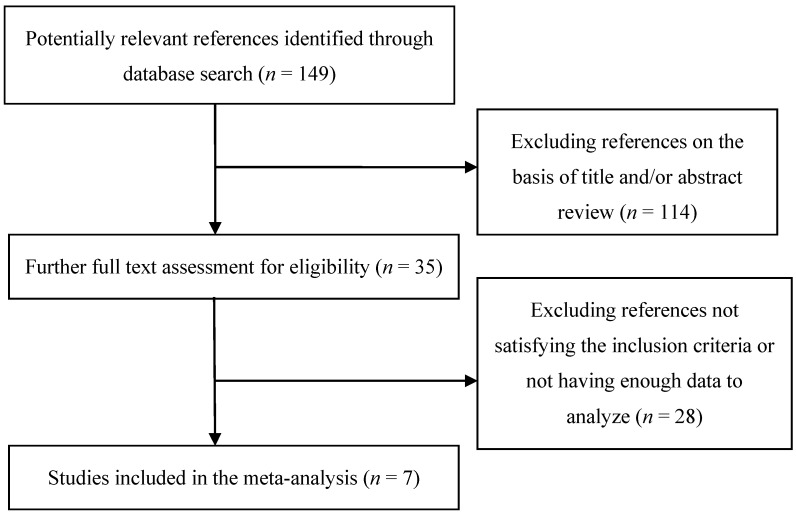
Flow diagram of literature search and identification of relevant studies.

The literature selection and identification flow diagram was shown in Figure 1. Out of 149 initially identified references through database search, 35 were obtained for further screen after initial titles and/or abstracts screening. Following full text assessment 28 references were then excluded for being mechanistic studies or not providing enough data. Finally, seven eligible studies comprising 5690 PD patients and 21251 matched controls were identified, which formed the basis of the present analysis [15,16,17,18,19,20,21]. The seven studies are all observational studies and the years of publication ranged from 2008 to 2015. Among the seven studies, five were conducted in the USA, one in China, and one in Japan.

### 3.2. Results of Meta-analysis

#### 3.2.1. Assessment of Vitamin D Level and Risk of PD

**Figure 2:**
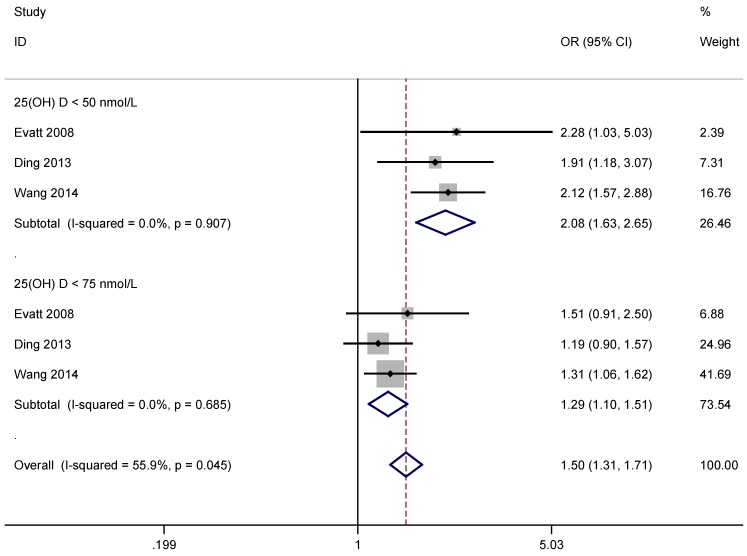
Forest plots of deficient and insufficient vitamin D levels and risk of Parkinson’s Disease (PD).

Three studies covering 966 PD patients and 813 age-matched controls were included in the quantitative analysis of vitamin D level and risk of PD [15,16,17]. Main descriptive data from the studies are shown in Table 1. Results of the meta-analysis revealed that low vitamin D level was associated with significantly increased risk of PD overall according to the OR: 1.50, 95% CI: 1.31 to 1.71. Vitamin D deficient individuals (serum 25(OH)D level <50 nmol/L) had a two-fold increased risk of PD in comparison with controls OR: 2.08, 95% CI: 1.63 to 2.65, and vitamin D insufficient individuals (serum 25(OH)D level <75 nmol/L) experienced a 30% increased risk of PD compared with controls (OR: 1.29, 95% CI: 1.10 to 1.51) (Figure 2). There was no evidence for significant statistical heterogeneity between the eligible studies.

#### 3.2.2. Assessment of Vitamin D Supplementation and Risk of PD

**Figure 3:**
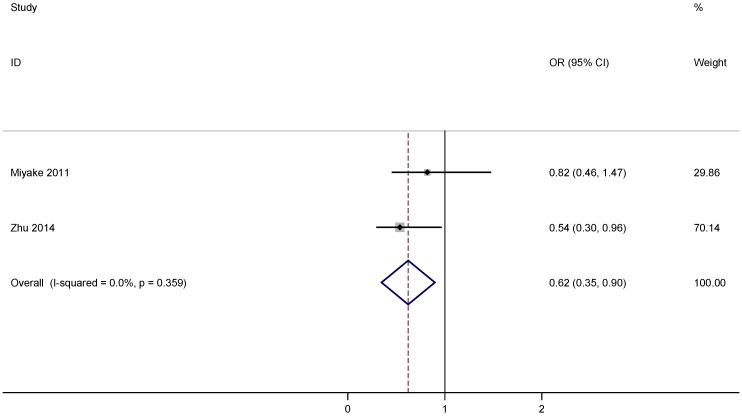
Forest plots of vitamin D supplementation and risk of PD.

Two case-control studies covering 458 patients with PD and 578 cases were included in the assessment of association between vitamin D supplementation and PD risk and the main descriptive data from the studies are shown in Table 2 [18,19]. As shown in Figure 3, the calculated OR for comparison of the highest with the lowest vitamin D supplementation was 0.62, 95% CI 0.35 to 0.90. This indicated that vitamin D supplementation was associated with a decreased risk of developing PD by 38%. No evidence for significant statistical heterogeneity was observed between the eligible studies.

#### 3.2.3. Assessment of Outdoor Work and Risk of PD

**Figure 4:**
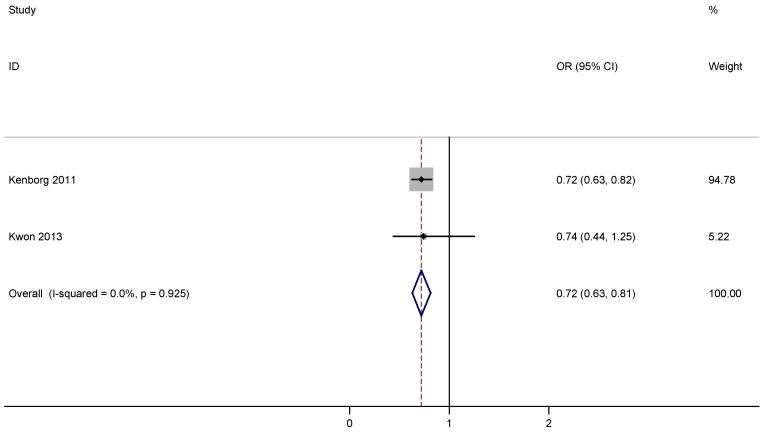
Forest plots of outdoor work and risk of PD.

There were 4266 PD patients and 19860 controls included in two eligible case-controls studies about the association of outdoor work and risk of PD [20,21]. Table 2 lists the main descriptive data from the eligible studies. A statistically significant inverse relation was found between outdoor work and risk of PD according to the pooled OR: 0.72, 95% CI: 0.63 to 0.81 (Figure 4), and there was no evidence for significant statistical heterogeneity.

**Table 1:** Main characteristics of eligible studies for the analysis of vitamin D deficiency and risk of PD.

References	Year	Country	Study Type	Number of Participants		Average Age (years)		OR (95% CI) *	Adjustments
				PD	Control	PD	Control		
Evatt [15]	2008	USA	cohort	100	99	65.4	65.7	2.28 (1.03, 5.03) (deficient) 1.51 (0.91, 2.50) (insufficient)	age, sex, race, symptom duration, and sampling season
Ding [16]	2013	USA	cross-sectional and case-control	388	283	65.7 ± 9.6	8.0 ± 10.4	1.91 (1.18, 3.07) (deficient) 1.19 (0.90, 1.57) (insufficient)	age, sex, race, and vitamin D supplementation
Wang [17]	2015	USA	case-control	478	431	64 ± 12	70 ± 8	2.12 (1.57, 2.88) (deficient) 1.31 (1.06, 1.62) (insufficient)	age, sex, and sampling season

* To unify the data OR and 95% CI were calculated using Stata according to the corresponding events and total numbers in PD and controls groups of eligible studies.

**Table 2:** Main characteristics of eligible studies for the analysis of vitamin D supplementation, outdoor work and risk of PD.

References	Year	Country	Study Type	Participants		Average Age (years)		OR, 95% CI High Versus Low Category	Adjustments
				PD	Control	PD	Control		
**Vitamin D supplementation**									
Miyake [18]	2011	Japan	case-control	249	368	68.5	66.6	0.82 (0.46–1.47)	age, sex, region of residence, pack-years of smoking, years of education, body mass index and dietary factors including cholesterol, dietary glycemic index, vitamin E, β-carotene, vitamin B6, caffeine, iron, and alcohol
Zhu [19]	2014	China	case-control	209	210	64.6 ± 9.4	66.0 ± 8.1	0.54 (0.30–0.96)	age, sex, smoking, alcohol use, education, and BMI
**Outdoor work**									
Kenborg [20]	2011	USA	case-control	3819	19,282	71.2	-	0.72 (0.63–0.82)	age, chronic obstructive pulmonary disease, comorbidity, place of birth, and social class
Kwon [21]	2013	USA	case-control	447	578	66	68	0.74 (0.44–1.25)	age, sex, and smoking

## 4. Discussion

Vitamin D is obtained through the diet and is photosynthesized in the skin upon exposure to solar ultraviolet B radiation. Vitamin D deficiency has been found to be associated with various chronic diseases conditions, such as cancer, cardiovascular diseases, diabetes, stroke, and neurodegenerative diseases, which aroused much interest for the potential of vitamin D in prevention and intervention of these disorders in recent years [1,2,3,4,5]. The lower vitamin D level in patients with PD compared with age-matched controls has been widely investigated [10,11,12,13,14]. Previous meta-analyses have consistently found that PD patients had lower levels of vitamin D than controls [11,12,13,14], while the associations between vitamin D status, supplementation, outdoor work and risk of PD merit to be further evaluated. To further comprehensively explore the association between vitamin D and PD, the present meta-analysis was designed to quantitatively assess the associations between vitamin D level and risk of PD, and vitamin D supplementation and outdoor work in lowering PD risk. Pooled data showed that subjects with deficient vitamin D level (25(OH)D < 50 nmol/L) experienced a two folds of PD risk (OR: 2.08, 95% CI: 1.63 to 2.65), and vitamin D insufficient subjects (25(OH)D < 75 nmol/L) also have increased PD risk according to the corresponding OR: 1.29, 95% CI: 1.10 to 1.51. This is in line with the suggestion that high vitamin D level exhibits protective effects against PD [22]. Both vitamin D supplementation and outdoor work were associated with a significantly reduced risk of PD indicated by the respective calculated OR: 0.62, 95% CI: 0.35 to 0.90, and OR: 0.72, 95% CI: 0.63 to 0.81.

The possible biological underpinning underlying the association between vitamin D level and PD risk should be multiple. First, as the primary mediator of vitamin D’s biological actions, the vitamin D receptor (VDR) exhibits multiple genetic polymorphisms, and many studies have reported the association between VDR polymorphisms and risk of PD [23,24,25]. Second, as oxidative stress contributes to the pathogenesis of PD [26], the attenuated antioxidant capacity of reduced level of vitamin D may increase the risk of PD. Third, vitamin D has been revealed to be crucial to active human T cells and vitamin D deficiency may prohibit T cell from clearing the insoluble α-synuclein fibrils and thus, may increase the PD risks [27,28]. Certainly, the mechanisms underlying the association between vitamin D level and PD risk is not limited to the above aspects and can be enriched by more mechanistic studies.

Pooled results of the meta-analysis should be considered in the context of its limitations. First, the number of the eligible studies and covered participants was relatively small. Second, the vitamin D supplementation and out-door activity are based on self-administered diet history questionnaire and self-report questionnaire. The confounders for which adjustments are made also varied in each study. These factors will potentially affect the results. Third, the potential effects of anti-PD drugs cannot be explicitly considered in the included studies. Fourth, due to the limited eligible studies we cannot perform gender and ethnic subgroup analysis.

## 5. Conclusions

In conclusion, based on the eligible studies we found that low vitamin D level was associated with increased risk of PD, which is consistent with the hypothesis that low vitamin D status is a risk factor of PD. In addition, it was indicated that vitamin D supplementation and outdoor work may reduce PD risk. Thus, optimizing vitamin D level may represent a potential avenue for the prevention of PD. As only a few studies have investigated the effects of vitamin D supplementation against PD so far, further research on this issue is strongly encouraged.
